# Intracardiac echocardiography versus transesophageal echocardiography guidance on left atrial appendage occlusion in patients with atrial fibrillation: A systematic review and meta‐analysis

**DOI:** 10.1002/joa3.70115

**Published:** 2025-07-01

**Authors:** Ubaid Khan, Ahmed Mazen Amin, Zuhair Majeed, Muhammad Haris Khan, Mahmoud Shaaban Abdelgalil, Muhammad Mubariz, Shrouk Ramadan, Muhammad Imran, Ahmed Raza, Muhammad Naveed Ur Rehman, Syed Hassnain Zafar Bukhari, Ahmad Talal Asif, Fahad Hassan Gunjial, Junaid Ali, Anum Nawaz

**Affiliations:** ^1^ Division of Cardiology University of Maryland School of Medicine Baltimore Maryland USA; ^2^ Faculty of Medicine Mansoura University Mansoura Egypt; ^3^ Department of Medicine King Edward Medical University Lahore Pakistan; ^4^ Department of Medicine Saidu Medical College Swat Pakistan; ^5^ Faculty of Medicine Ain Shams University Cairo Egypt; ^6^ Department of Medicine Akhtar Saeed Medical College Lahore Pakistan; ^7^ Department of Medicine, University College of Medicine and Dentistry The University of Lahore Lahore Pakistan; ^8^ Department of Medicine Services Institute of Medical Sciences Lahore Pakistan; ^9^ Department of Medicine Mayo Hospital Lahore Pakistan; ^10^ Clinical Development Fellow King's Mill Hospital Sherwood Forest Hospitals NHS Foundation Trust Nottinghamshire UK; ^11^ Department of Medicine Saint Peter's University Hospital New Brunswick New Jersey USA; ^12^ Department of Radiology Pakistan Atomic Energy Commission Hospital Islamabad Pakistan

**Keywords:** anticoagulation, atrial fibrillation, intracardiac echocardiography, meta‐analysis, transesophageal echocardiography

## Abstract

**Background:**

Intracardiac echocardiography (ICE) is an innovative technique that has emerged as an alternative to transesophageal echocardiography (TEE) to guide the implantation of a left atrial appendage occlusion (LAAO) device in patients with nonvalvular atrial fibrillation (AF) who cannot tolerate anticoagulants.

**Purpose:**

We aim to review the clinical efficacy and safety of ICE compared to TEE to guide the implantation of LAAO devices in patients with AF.

**Methods:**

We conducted comprehensive searches across PubMed, CENTRAL, Web of Science, Scopus, and EMBASE until March 2024. Pooled data were reported using risk ratio (RR) for dichotomous outcomes and mean difference (MD) for continuous outcomes, along with a 95% confidence interval (CI). This systematic review and meta‐analysis was registered with PROSPERO ID: CRD42024542537.

**Results:**

We included 19 studies involving 44,706 patients. ICE was associated with a statistically significant high procedure success rate compared to TEE (RR: 1.0055 with 95% CI [1.0006, 1.0104], *p* = 0.01), but there was no difference in procedure duration (MD: 3.07 with 95% CI [−4.67, 10.80], *p* = 0.44) between the two groups. However, compared to the ICE group, patients undergoing LAAO under TEE guidance required more than one device more often (RR: 1.39 with 95% CI [1.23, 1.57], *p* < 0.01). The TEE group also reported a reduced incidence of pericardial effusion compared to the ICE group (RR: 0.65 with 95% CI [0.50, 0.85], *p* < 0.01).

**Conclusion:**

Our meta‐analysis concluded that ICE can be a viable alternative to TEE for guiding LAAO, particularly in patients unsuitable for general anesthesia. It can also reduce the need for GA and adverse effects and resources associated with it, require fewer devices, and demonstrate comparable safety and efficacy outcomes, though it may increase the risk of pericardial effusion. Further prospective trials are warranted.

## Graphical abstract

ICE is a viable alternative to TEE for guiding LAAO in atrial fibrillationpatients unable to tolerate anti‐coagulants, especially those unfit for general anesthesia. It offers high procedural success, reduced device use, and comparable safety, though with a slightly increased risk of pericardial effusion.

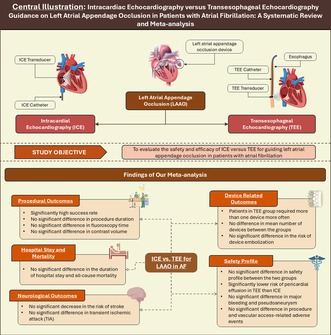

## 1 INTRODUCTION

Left atrial appendage occlusion (LAAO) has emerged as a promising strategy for stroke prevention in high‐risk patients with nonvalvular atrial fibrillation (AF).^1^ This minimally invasive procedure aims to close off the left atrial appendage (LAA), a small pouch in the heart where blood clots are more likely to form. By sealing the LAA, the risk of these clots traveling downstream and causing a stroke is significantly reduced.^2^

Accurate device positioning and assessment of peri‐procedural complications are crucial for successful LAAO.^3^ Traditionally, transesophageal echocardiography (TEE) has been the gold standard imaging modality for LAAO guidance.^4^ TEE provides detailed anatomical information using an ultrasound probe inserted through the esophagus.^5^ However, TEE requires general anesthesia (GA), which carries inherent risks and prolongs procedure time.^6^

Intracardiac echocardiography (ICE) offers a potential alternative to TEE.^7^ ICE is a catheter‐based imaging technique that provides real‐time visualization of the left atrium during LAAO.^8^ Its use does not require GA and may enhance procedural efficiency.^9^ Unlike TEE, ICE uses a miniaturized probe directly positioned within the heart chamber, offering a more magnified view of the LAA and surrounding structures.^6^ Numerous studies have established the feasibility of employing ICE guidance for LAAO in both single‐center and multicenter settings.^10^ Yet, the adoption of ICE in US LAAO practices has remained low due to the learning curve, limitations of 2D‐ICE, limited formal education programs, and the lack of consensus on optimal imaging methodologies.^11^

So, the comprehensive understanding of ICE performance compared to TEE remains elusive. However, the emergence of new 3D and 4D ICE technologies has revolutionized real‐time imaging during procedures, reigniting interest in using ICE to guide LAAO procedures, especially during the COVID‐19 pandemic. With continuous advancements in ICE technology, ICE is becoming a crucial imaging tool for guiding an increasing number of LAAO cases worldwide.^12^ New device iterations and increasing operator experience with ICE necessitate an updated assessment of both techniques. Our study aims to address this gap by conducting a comprehensive, up‐to‐date meta‐analysis comparing the efficacy and safety of TEE and ICE for LAAO procedures. We will investigate key outcomes such as procedural success, efficacy (time and resource utilization), and safety profiles (peri‐procedural and device‐related complications).

## 2 METHODOLOGY

### 2.1 Protocol registration

The present systematic review and meta‐analysis followed the guidelines outlined in the Preferred Reporting Items for Systematic Reviews and Meta‐Analyses (PRISMA)^13^ and the Cochrane Handbook of Systematic Reviews and Meta‐Analysis.^14^ The study protocol was registered with the International Prospective Register of Systematic Reviews (PROSPERO) under the registration PROSPERO ID: CRD42024542537.

### 2.2 Data sources and search strategy

Until March 2024, a comprehensive search was systematically conducted across five databases (PubMed, CENTRAL, Web of Science, Scopus, EMBASE) by U.K. without imposing any search restrictions. Detailed information about the search strategy is available in (Table S1).

### 2.3 Eligibility criteria

We included observational comparative studies if they met the following PICO criteria: (P) patients with AF undergoing LAAO, (I) LAAO under ICE guidance, (C) LAAO under TEE guidance, and (O) procedural success, fluoroscopy time, contrast volume, and number of devices.

We excluded animal studies, reviews, pilot studies, protocols, conference abstracts, editorial articles, and book chapters.

### 2.4 Study selection

Search results from all the databases were imported to Covidence.org, and duplicates were removed automatically. Four authors screened the remaining records independently (S.R., A.M.A., M.I., and F.H.G), and any conflict between them was resolved by another author (U.K). The screening was done in two steps: (I) title and abstract screening to determine the study's relevance for this meta‐analysis, and (ii) full‐text screening according to the inclusion criteria for the final eligibility for qualitative and quantitative analysis.

### 2.5 Data extraction

Data were collected independently by six review authors (M.M., M.S., S.R., M.N.R., J.A., and M.H.K) and extracted into a uniform data extraction Excel sheet. The extracted data included characteristics of the included studies, including first author name, year of publication, country, study design, total participants, type of device used, inclusion criteria, primary outcome and follow‐up duration; participants' baseline characteristics, including the number of participants, mean age, gender, AF types, HAS‐BLED score, CHA2DS2‐VASc score and associated comorbidities; and outcome measures as previously described across the intervention and comparator group. Any disagreement was resolved by consensus.

### 2.6 Risk of bias and certainty of evidence

The quality assessment of studies was independently conducted using the Cochrane ROBINS‐I tool^15^ by (A.N., U.K., Z.M., F.H.G., S.H.Z.B., and A.T.A). Moreover, they evaluated seven domains, including bias due to confounding, bias in the selection of participants into the study, bias in classification of intervention, bias due to deviation from intended interventions, bias due to missing data, bias in measurement of outcomes, and bias in selection of reported results. Any conflicts have been resolved by consensus and discussion.

### 2.7 Statistical analysis

We conducted a statistical analysis using R software version 4.3.1. The analysis combined results from multiple studies using either risk ratios (RR) (for dichotomous outcomes) or mean differences (MD) (for continuous outcomes), both with 95% confidence intervals. A random effects model was applied when significant heterogeneity (*I* ^2^ > 50%) was detected using the chi‐square and *I*‐square tests; otherwise, a common effect model was used. Heterogeneity was interpreted according to the Cochrane Handbook (chapter nine),^16^ with an *I* ^2^ value of 0–40 percent indicating low heterogeneity, 30–60 percent signifying moderate heterogeneity, 50–90 percent may represent substantial heterogeneity, and 75–100 percent signifying considerable heterogeneity. A chi‐square test *p*‐value below 0.1 was considered statistically significant for heterogeneity.

## 3 RESULTS

### 3.1 Search results and study selection

**FIGURE 1:**
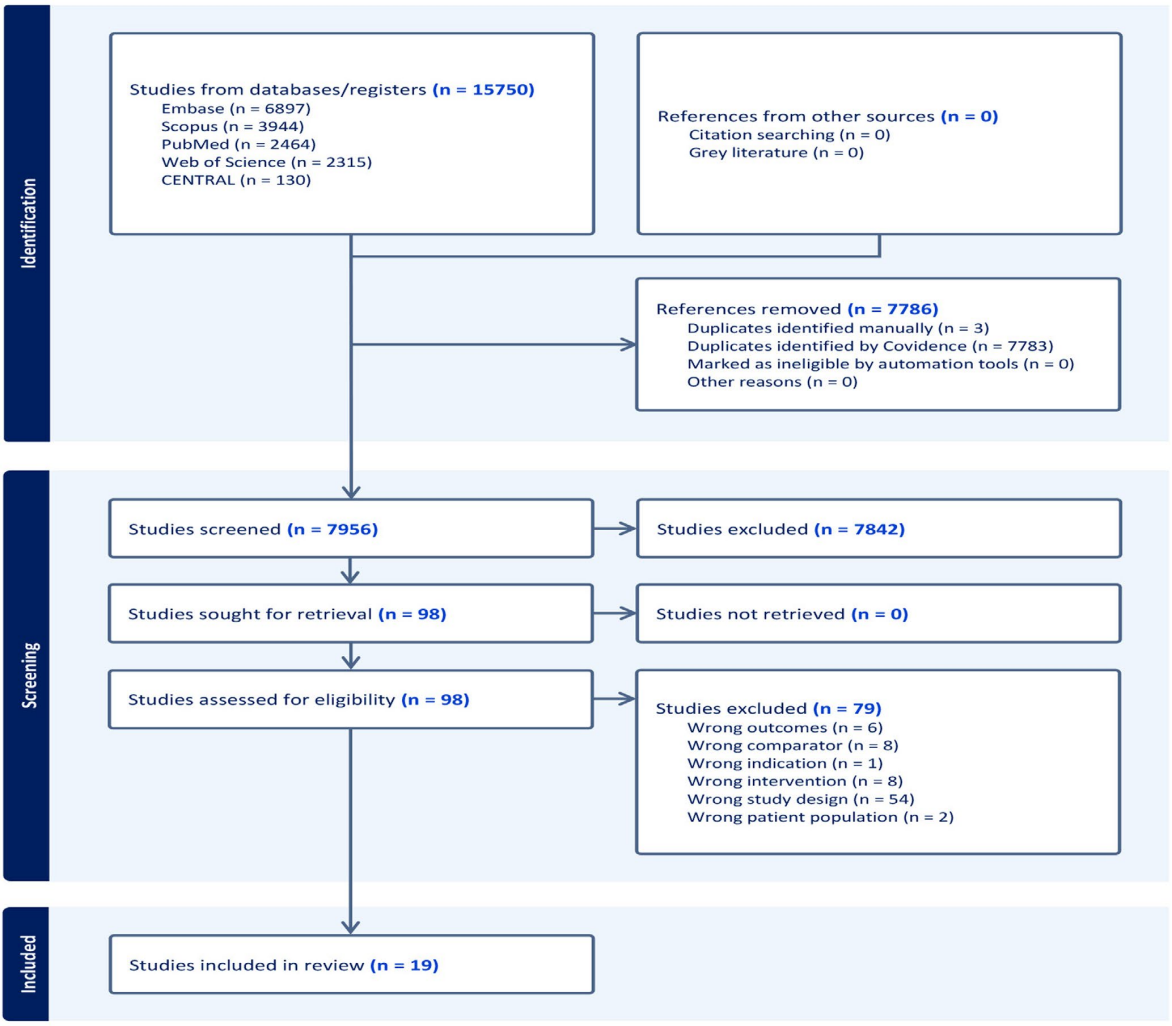
PRISMA flow chart of the screening process.

A total of 15,750 studies were incorporated from five databases into Covidence. Covidence removed 7786 duplicates, leaving 7956 records for screening. Of these, 7842 records were deemed irrelevant and excluded during the title and abstract screening process. This left 98 studies for full‐text screening, and 19 were found to be eligible for data extraction (Figure 1).

### 3.2 Study characteristics

The included studies comprised 19 prospective cohort studies,^17^, ^18^, ^19^, ^20^, ^21^, ^22^, ^23^, ^24^, ^25^, ^26^, ^27^, ^28^, ^29^, ^30^, ^31^, ^32^, ^33^, ^34^, ^35^ with a total of 44,706 patients. Among these, 39,102 patients were in the TEE group, and 5604 were in the ICE group. The devices utilized were WATCHMAN, Amplatzer Cardiac Plug (ACP) or Amulet, and LAmbre. The patients were 18 years and older, with a mean age of approximately 71.6 years; 59.43% of the participants were male. Detailed characteristics of the included studies and study participants are presented in Tables 1 and 2, respectively.

**TABLE 1:** Shows study characteristics.

Study ID	Design	Country	Number of centers	Number of participants	Main inclusion criteria	Type of the device used	Follow‐up duration (months)	Primary outcome
Pastormerlo et al. 2023^29^	Prospective cohort	Italy	26	772	Patients with nonvalvular atrial fibrillation, a CHA2DS2‐VASc score ≥2 and relative/absolute contraindication for NOACs	Watchman	12	Major adverse events
Shang et al. 2023^35^	Prospective cohort	China	1	302	Adults (>18 years) with documented paroxysmal or persistent nonvalvular atrial fibrillation refractory to antiarrhythmic drugs, CHA2DS2‐VASc score ≥2 in men or ≥3 in women, and at least one of the following: high bleeding risk (HAS‐BLED score ≥3), contraindications for long‐term oral anticoagulation, intolerance/refusal of oral anticoagulation, or preference for Watchman device implantation as an alternative to long‐term oral anticoagulation	Watchman	16.1 ± 3.1	Procedural characteristics
Ferro et al. 2023^21^	Prospective cohort	United States	698	34,107	Patients who underwent LAAO procedures with a Watchman FLX device from August 2020 to September 2021. Both successful and aborted procedures (where venous access was obtained but the device was not deployed) were included to enhance the study's generalizability	Watchman	1.5	Procedural characteristics and adverse events
Grazina et al. 2023^23^	Retrospective cohort	Portugal	1	88	Atrial fibrillation patients who underwent left atrial appendage occlusion between 2009 and 2020, had a CHA2DS2‐VASc score ≥2, and either had contraindications to or experienced inefficacy of oral anticoagulants. Some patients were selected for LAAO with intracardiac echocardiography guidance based on clinical factors	Amulet/ACP/Watchman/LAmbre	19 ± 10	Major adverse events
Zahid et al. 2022^34^	Retrospective cohort	United States	NA	2820	National Inpatient Sample for all hospitalizations for left atrial appendage closure (LAAC) from Q4 of 2015 to 2019 were identified using ICD‐10 code of 02L73DK	NA	NA	Adverse events
Su et al. 2022^33^	Prospective cohort	China	39	2603	Patient of 18 years or older, eligible for a WATCHMAN device according to current guidelines and physician discretion, and capable of providing informed consent for all related procedures at an approved clinical center	Watchman	60	Technical success
Streb et al. 2019^32^	Prospective cohort	Poland	1	23	Patients diagnosed with atrial fibrillation, echocardiographic eligibility for LAAO confirmed by TOE, increased stroke risk (CHA_2_DS_2_‐VASc score ≥2), contraindications for oral anticoagulation, and provision of informed consent	Amulet	1	Adverse events
Reis et al. 2018^31^	Prospective cohort	Portugal	1	82	Patients with nonvalvular atrial fibrillation, high embolic risk (CHA2DS2VASc score ≥2), and ineligibility for oral anticoagulation were included	Amulet/ACP/Watchman	23 ± 1	Adverse events
Pommier et al. 2021^30^	Prospective cohort	France	1	224	Patients with nonvalvular atrial fibrillation (chronic, persistent, or paroxysmal) were included if they had an elevated stroke risk (high CHA2DS2‐VASc score) and bleeding risk (high HAS‐BLED score). Eligibility also required an absolute or relative contraindication for oral anticoagulation	ACP/Watchman	15 ± 18	Adverse events
Nielsen‐Kudsk et al. 2019^28^	Prospective cohort	Global	75	1085	Patients with atrial fibrillation at high risk for stroke and bleeding who were eligible for left atrial appendage occlusion with the Amplatzer Amulet device	Amulet	24	Major adverse events
Morcos et al. 2021^27^	Retrospective cohort	United States	NA	790	Adults who underwent LAAC procedures under TEE or ICE guidance	Amulet/Watchman	NA	Major adverse events.
Korsholm et al. 2017^26^	Retrospective cohort	Denmark	1	216	Patients undergoing LAAO procedure using either the Amplatzer Cardiac Plug or Amulet device	ACP/Amulet	1.9 ± 0.4	Technical success
Kim et al. 2018^25^	Retrospective cohort	Korea	2	144	Patients with nonvalvular atrial fibrillation who had a high risk of stroke (CHA2DS2‐VASc score ≥2), contraindications for long‐term oral anticoagulant therapy, or recurrent AF‐related strokes despite appropriate anticoagulation	Amulet/ACP/Watchman	24	Procedure‐related complications
Hemam et al. 2019^24^	Prospective cohort	United States	3	104	Patients with nonvalvular atrial fibrillation, significant stroke risk, and a history of bleeding or contraindication for long‐term anticoagulation who were referred for left atrial appendage closure	watchman	2.8 ± 1.2	Procedural parameters
Gianni et al. 2021^22^	Prospective cohort	United States	1	190	Patients were eligible if they had LAAO performed as a standalone procedure, following ablation of extra‐LAA sites, or after failed Lariat epicardial ligation	Watchman	2.0 ± 1.5	Peri‐procedural and device‐related complications
Chen et al. 2022^19^	Prospective cohort	China	1	190	Patients age >18 years, CHA2DS2‐VASc score ≥2, contraindication to long‐term oral anticoagulant therapy, bleeding events with or without anticoagulation, stroke despite oral anticoagulation, and intolerance or refusal to take oral anticoagulants	LAmbre	3	Procedural parameters and complications
Berti et al. 2018^18^	Retrospective cohort	Italy	16	604	Patients with paroxysmal, persistent or permanent nonvalvular atrial fibrillation (NVAF), a CHA2DS2‐VASc score ≥2, and contraindication for oral anticoagulants (OACs) or previous stroke during treatment with OACs	ACP/Amulet	16.8 ± 18.3	Procedural results and in‐hospital outcomes
Alkhouli et al. 2020^17^	Prospective cohort	United States	1	286	Patients were eligible for inclusion if they had LAAO performed with either ICE or TEE guidance. Importantly, the study did not apply any pre‐specified anatomic or clinical exclusion criteria, aiming to assess the utility of “routine” ICE‐guided LAAO in all comers	Watchman	1.5 ± 0.5	Technical success, procedural complications
Frangieh et al. 2016	Prospective cohort	Switzerland	1	76	Patients with nonvalvular atrial fibrillation, elevated stroke risk (CHA2DS2‐VASc score), and bleeding risk (HAS‐BLED score), who had contraindications for or reasons to avoid oral anticoagulation	Watchman	NA	Adverse events

**TABLE 2:** shows: Baseline characteristics of included patients.

Study ID	Number of patients		Mean age in years (SD)		Males (%)		Atrial fibrillation				HAS‐BLED score		CHA2DS2‐VASc score		Comorbidities *N* (%)							
							Paroxysmal AF (%)		Persistent AF (%)						HTN (%)		DM (%)		HF		Previous stoke/TIA	
	TEE	ICE	TEE	ICE	TEE	ICE	TEE	ICE	TEE	ICE	TEE	ICE	TEE	ICE	TEE	ICE	TEE	ICE	TEE	ICE	TEE	ICE
Pastormerlo et al. 2023^29^	623	149	65.0 (8.5)	77.0 (7.5)	407 (65.0)	97 (65.0)	NA	NA	304 (48)	72 (48)	3.7 (1.1)	3.5 (1.4)	4.1 (1.4)	4.2 (1.8)	491 (78.0)	115 (77.0)	219 (34.0)	45 (30.0)	NA	NA	87 (13)	19 (12)
Shang et al. 2023^35^	109	193	64.2 (7.8)	65.0 (8.5)	69 (63.3)	109 (56.4)	44 (40.37)	95 (49.22)	65 (59.6)	98 (50.8)	2.07 (1.28)	2.19 (1.15)	3.41 (1.82)	3.87 (1.60)	71 (65.1)	114 (59.1)	27 (24.8)	61 (31.6)	39 (35.78)	79 (40.93)	46 (42.20)	98 (50.78)
Ferro et al 2023^21^	31,835	2272	76.4 (7.9)	75.8 (8.0)	18,817 (59.1)	1365 (60.1)	19,735 (62.5)	1257 (55.7)	6076 (19.2)	599 (26.5)	2.4 (1.0)	2.5 (1.0)	4.8 (1.5)	4.8 (1.5)	29,194 (91.7)	2083 (91.7)	11,350 (35.7)	837 (36.9)	12,449 (39.1)	791 (34.8)	6851 (21.5)	524 (23.1)
Grazina et al. 2023^23^	43	45	74.2 (9.8)	75.5 (9.6)	28 (65.1)	32 (71.1)	NA	NA	32 (74.4)	31 (68.9)	3.6 (1.0)	3.6 (1.1)	4.1 (1.4)	4.0 (1.4)	36 (83.7)	31 (68.9)	13 (30.2)	15 (33.3)	NA	NA	NA	NA
Zahid et al. 2022^34^	1410	1410	75.0 (7.4)	74.3 (7.4)	895 (63.5)	870 (61.7)	NA	NA	NA	NA	NA	NA	NA	NA	1195 (84.8)	1215 (86.2)	255 (18.1)	290 (20.6)	20,910 (34.5)	415 (29.4)	NA	NA
Su et al. 2022^33^	2508	95	NA	NA	NA	NA	NA	NA	NA	NA	NA	NA	NA	NA	NA	NA	NA	NA	NA	NA	NA	NA
Streb et al. 2019^32^	12	11	73.0 (12.6)	77.0 (5.9)	4 (33.3)	5 (45.4)	8 (66.66)	6 (45.45)	NA	NA	2 [0.5]	3^1^	5 [1.5]	5 [2]	11 (91.7)	9 (81.8)	3 (25.0)	3 (27.3)	4 (33.3%)	2 (18.2%)	3 (25%)	4 (36.4%)
Reis et al 2018^31^	56	26	74.0 (8.0)	74.2 (7.9)	53 (9.6)	20 (76.9)	25 (30.5)	NA	4 (4.9)	0	3.3 (1.0)	3.3 (1.0)	4.25 (1.40)	4.27 (1.40)	48 (86.6)	20 (79.8)	26 (31.7)	6 (23.4)	NA	NA	34 (41.5%)	34 (41.5%)
Pommier et al. 2021^30^	49	175	75.0 (7.0)	76.0 (8.0)	35 (73.0)	122 (70.0)	11 (23)	51 (29)	37 (77.0)	122 (7.0)	3.93 (1.02)	4.07 (0.99)	4.5 (1.49)	4.2 (1.38)	46.0 (96.0)	160 (91.0)	10 (21.0)	60 (34.0)	8 (17%)	30 (17%)	31 (64%)	122 (70%)
Nielsen‐Kudsk et al. 2019^28^	955	130	75.0 (9.0)	75.0 (8.0)	620 (65.0)	78 (60.0)	NA	NA	NA	NA	3.3 (1.1)	3.2 (0.9)	4.2 (1.6)	4.1 (1.6)	NA	NA	NA	NA	NA	NA	35 (3.7%)	54 (41.5%)
Morcos et al 2021^27^	395	395	70.4 (12.9)	70.7 (12.7)	270 (68.4)	235 (59.5)	184 (46.7)	158 (40)	NA	NA	NA	NA	NA	NA	219 (55.4)	239 (59.2)	66 (16.8)	68 (17.1)	5 (1.3)	10 (2.5)	NA	NA
Korsholm et al. 2017^26^	107	109	73.0 (9.7)	73.0 (7.8)	79 (73.8)	68 (62.4)	45 (42)	52 (48)	8 (8.0)	7 (6.0)	4.1 (1.1)	4.1 (0.9)	4.4 (1.6)	4.1 (1.6)	86 (80.0)	91 (83.3)	23 (22.0)	23 (21.0)	21 (20%)	16 (15%)	50 (46%)	59 (55%)
Kim et al. 2018^25^	103	41	72.3 (9.2)	71.4 (9.3)	51 (49.5)	24 (58.5)	28 (27.2)	14 (34.1)	NA	NA	3.1 (1.4)	3.0 (1.5)	4.3 (1.4)	4.3 (1.4)	86 (83.5)	37 (90.2)	26 (25.2)	11 (26.8)	41 (39.8%)	18 (43.9%)	44 (42.7%)	20 (48.8%)
Hemam et al. 2019^24^	51	53	76.0 (7.0)	77.0 (10.0)	20 (39.2)	20 (37.7)	NA	NA	NA	NA	NA	NA	4.5 (1.6)	4.5 (1.8)	46 (90.0)	43 (81.0)	15 (29.0)	18 (34.0)	13 (25%)	10 (19%)	17 (33%)	22 (42%)
Gianni et al 2021^22^	68	122	75.0 (9.0)	72.0 (8.0)	41 (60.3)	81 (66.4)	NA	NA	NA	NA	2.7 (1.2)	2.7 (1.3)	4.3 (1.3)	4.1 (1.4)	NA	NA	NA	NA	NA	NA	NA	NA
Chen et al 2022^19^	121	69	70.8 (7.3)	73.0 (8.3)	80 (66.1)	50 (72.5)	NA	NA	NA	NA	2.9 (1.1)	2.6 (1.0)	4.4 (1.0)	4.4 (1.7)	100 (82.6)	49 (71.0)	34 (28.1)	19 (27.5)	NA	NA	68 (56.2)	37 (53.6)
Berti et al. 2018^18^	417	187	74.0 (7.0)	76.0 (8.0)	382 (91.6)	153 (81.8)	NA	NA	64 (15.3)	68 (36.4)	3.15 (1.10)	3.25 (1.00)	4.25 (1.40)	4.27 (1.40)	NA	NA	NA	NA	NA	NA	NA	NA
Alkhouli et al. 2020^17^	196	90	75.2 (7.8)	75.7 (8.0)	109 (55.6)	56 (62.2)	NA	NA	NA	NA	2.9 (1.1)	2.8 (1.2)	4.8 (1.6)	4.7 (1.4)	171 (87.2)	83 (92.2)	86 (43.9)	30 (33.3)	95 (48.5%)	51 (56.7%)	84 (42.9%)	33 (36.5%)
Frangieh et al 2016^20^	44	32	80.3 (7.7)	74.7 (9.3)	25 (57.0)	26 (81.0)	NA	NA	20 (46.0)	10 (31.0)	3.6 (1.4)	3.3 (0.8)	4 (1.5)	4.2 (2.2)	38 (86.0)	27 (84.0)	16 (36.0)	14 (44.0)	NA	NA	9 (20.5%)	9 (28.1%)

### 3.3 Quality assessment

**FIGURE 2:**
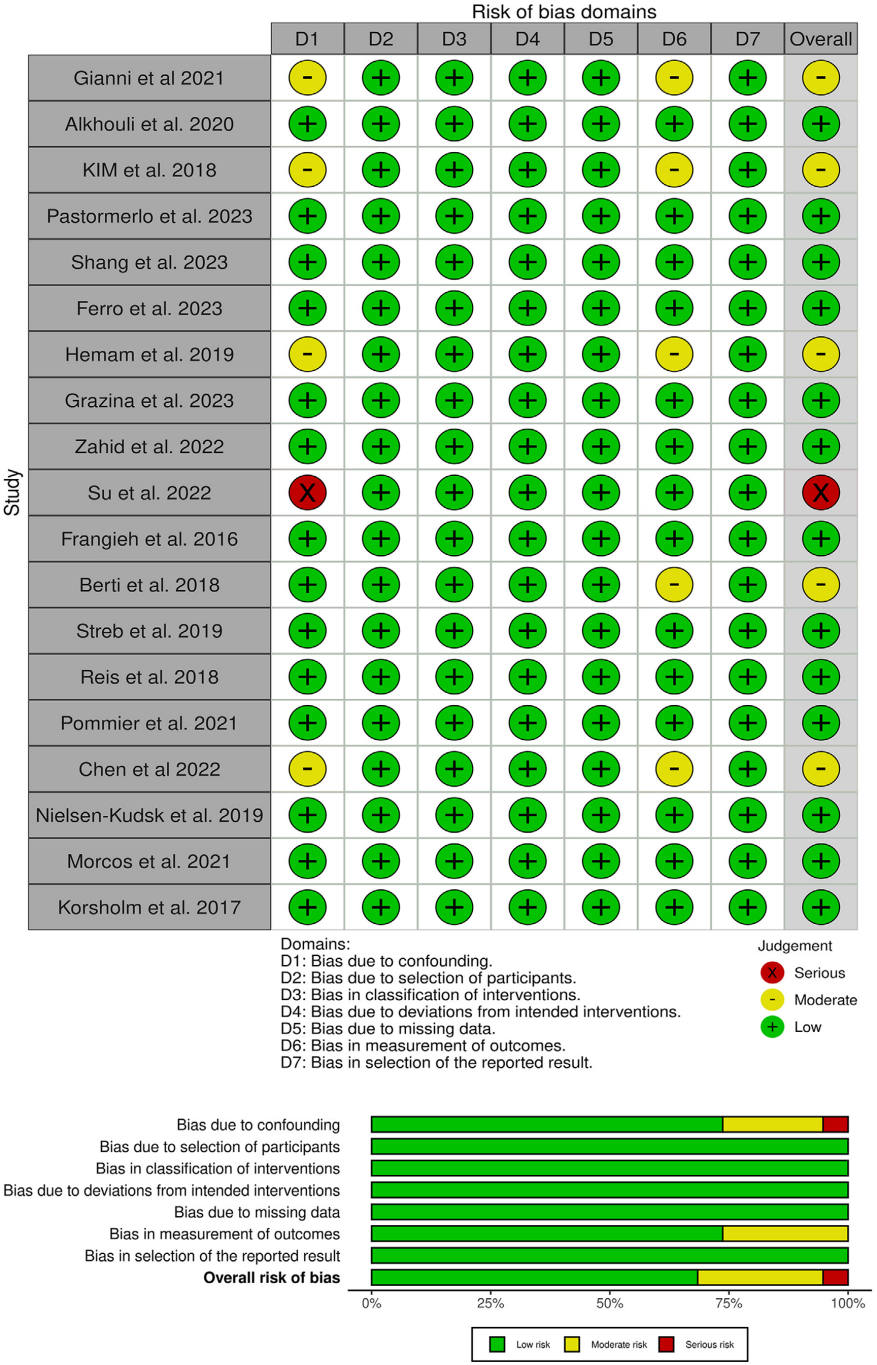
Quality assessment of risk of bias in the included trials. The upper panel presents a schematic representation of risks (low = green, unclear = yellow, and high = red) for specific types of biases of each study in the review. The lower panel presents risks (low = green, unclear = yellow, and high = red) for the subtypes of biases of the combination of studies included in this review.

ROBINS‐I assessment showed that Su et al. 2022 had an overall high risk of bias. However, Gianni et al. 2021, KIM et al. 2018, Hemam et al. 2019, Berti et al. 2018, and Chen et al. 2022 had some concerns overall, while the rest of the studies had an overall low risk of bias (Figure 2).

### 3.4 Primary outcomes

**FIGURE 3:**
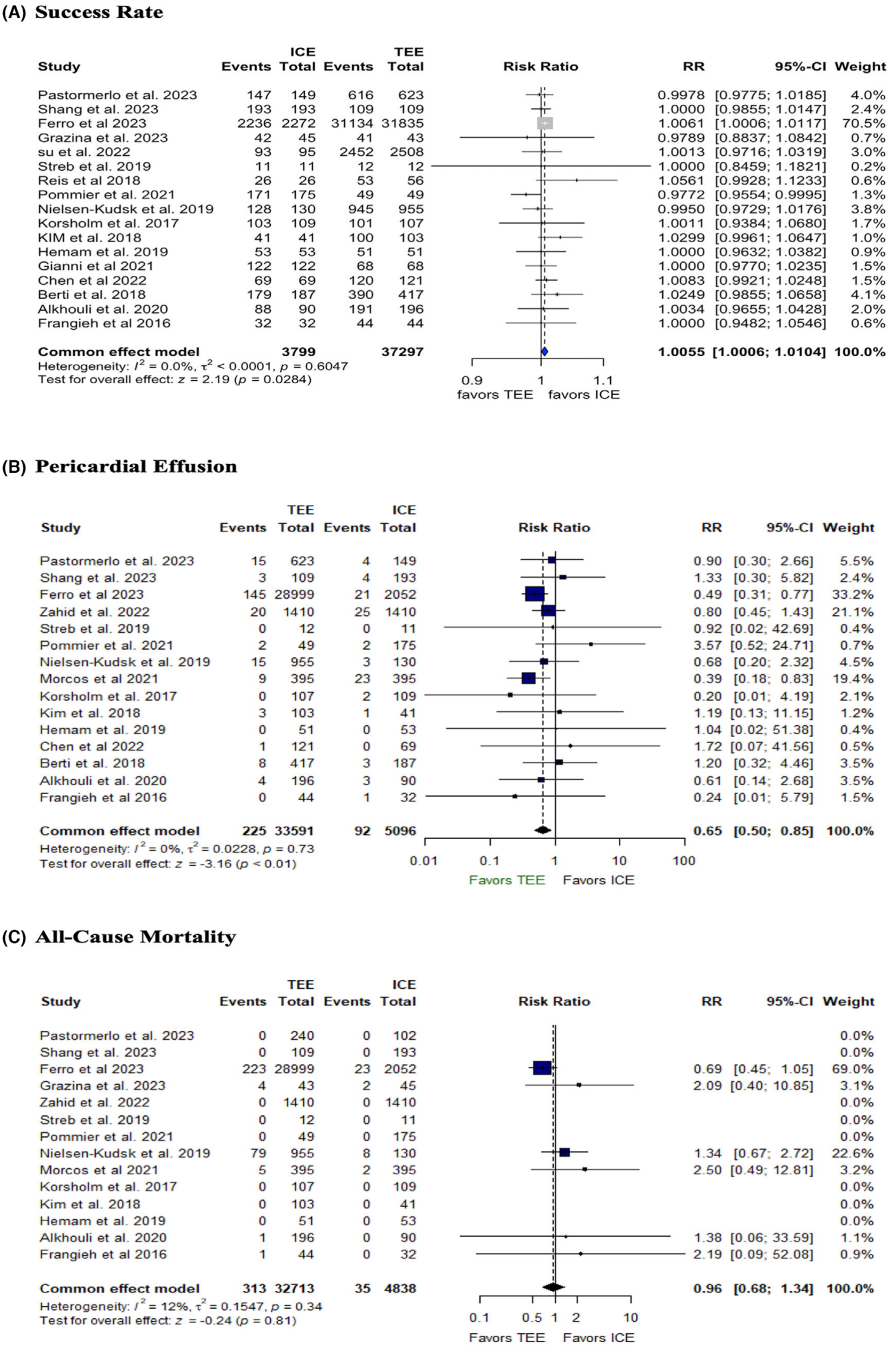
Forest plots of (A) success rate, (B) pericardial effusion, (C) all‐cause mortality. CI, confidence interval; RR, risk ratio.

Compared to TEE, ICE was associated with a statistically significant high success rate (RR: 1.0055 with 95% CI [1.0006, 1.0104], *p* = 0.01, *I* ^2^ = 0) (Figure 3A), though it may not be clinically significant. Additionally, TEE was associated with lower risk of pericardial effusion compared to ICE (RR: 0.65, 95% CI: [0.50, 0.85], *p* = 0.01, *I* ^2^ = 0) (Figure 3B), while there was no difference in all‐cause mortality between the two groups (RR: 0.96, 95% CI: [0.68, 1.34], *p* = 0.81, *I* ^2^ = 12) (Figure 3C).

### 3.5 Secondary outcomes

#### 3.5.1 Efficacy outcomes

**FIGURE 4:**
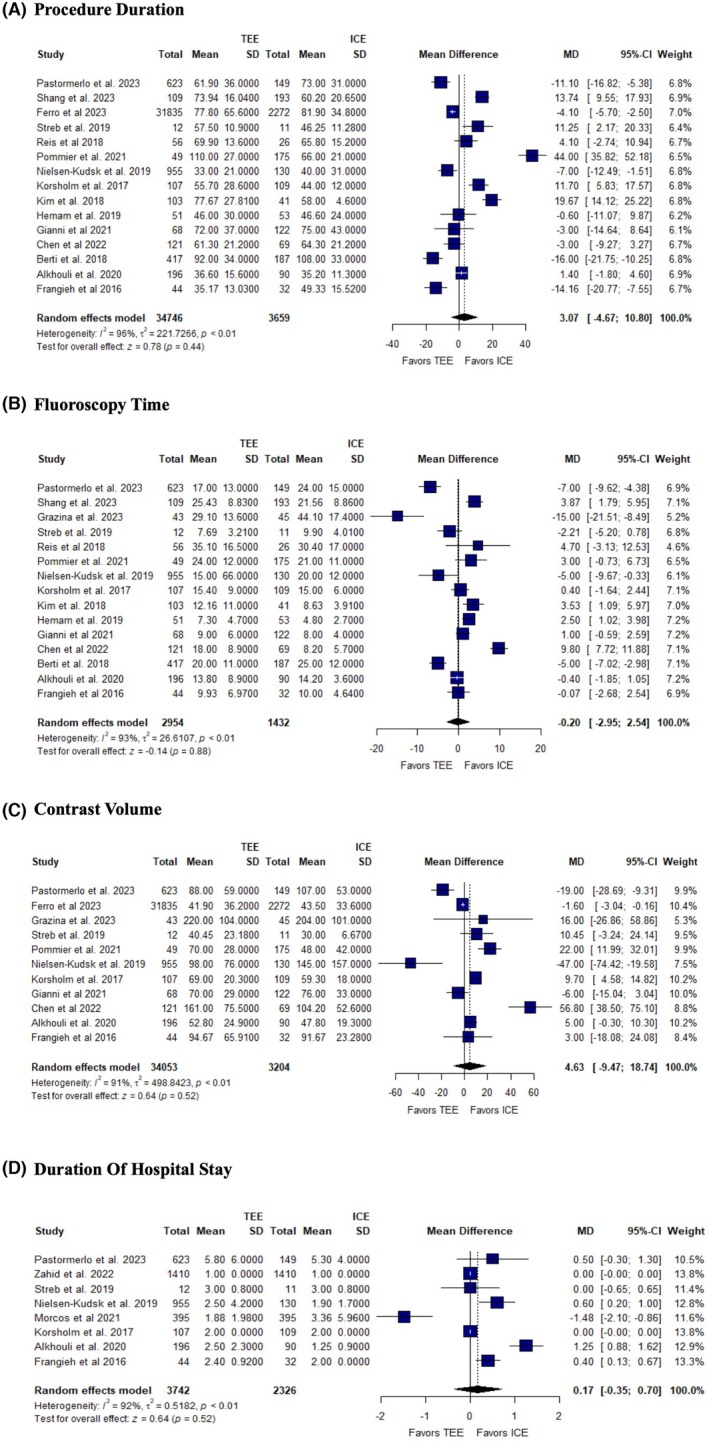
Forest plots of (A) procedure duration, (B) fluoroscopy time, (C) contrast volume, (D) duration of hospital stay. CI, confidence interval; MD, mean difference.

The pooled analysis showed no significant difference between TEE and ICE in procedure duration (MD: 3.07, 95% CI: [−4.6, 10.80], *p* = 0.44, *I* ^2^ = 96%) (Figure 4A), fluoroscopy time (MD: −0.20, 95% CI: [−2.95, 2.54], *p* = 0.88, *I* ^2^ = 93%) (Figure 4B), contrast volume (MD: 4.63, 95% CI: [−9.47, 18.74], *p* = 0.52, *I* ^2^ = 87%) (Figure 4C), and duration of hospital stay (MD = 0.17, 95% CI: [−0.35, 0.70], *p* = 0.52, *I* ^2^ = 92) (Figure 4D).

High heterogeneity was observed in procedure duration (*I* ^2^ = 96%), fluoroscopy time (*I* ^2^ = 93%), contrast volume (*I* ^2^ = 91%), and length of hospital stay (*I* ^2^ = 92). The leave‐one‐out analysis did not resolve the heterogeneity in these outcomes (Figures S1–S4), respectively.

**FIGURE 5:**
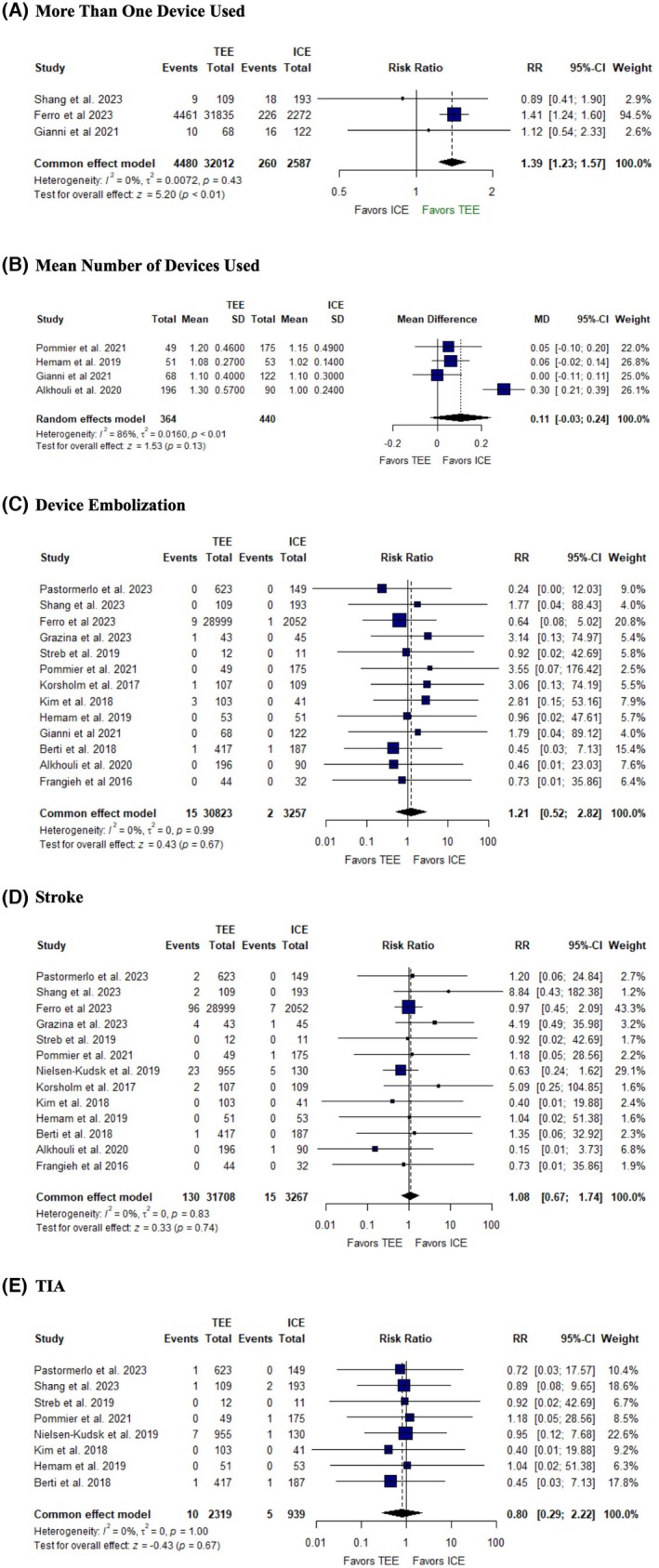
Forest plots of (A) more than one device used, (B) mean number of devices used, (C) device embolization, (D) stroke, (E) TIA. CI, confidence interval; MD, mean difference; RR, risk ratio.

The pooled analysis showed that patients in the TEE group required more than one device more often compared to the ICE group (RR: 1.39, 95% CI: [1.23, 1.57], *p* = 0.01, *I* ^2^ = 0%) (Figure 5A), while there was no difference in the mean number of devices used between groups (MD = 0.11, 95% CI: [−0.03, 0.24], *p* = 0.13, *I* ^2^ = 86%) (Figure 5B). There was no significant difference in the risk of device embolization between ICE and TEE groups (RR: 1.21, 95% CI: [0.52, 2.82], *p* = 0.67, *I* ^2^ = 0%) (Figure 5C).

Additionally, there was no significant difference in the risk of the stroke (RR: 1.08, 95% CI: [0.67, 1.74], *p* = 0.74, *I* ^2^ = 0%) (Figure 5D), and TIA (RR: 0.80, 95% CI: [0.29, 2.22], *p* = 0.67, *I* ^2^ = 0%) (Figure 5E) between the two groups.

The studies were homogenous in all outcomes, except high heterogeneity was observed in the mean number of devices (*I* ^2^ = 86%), which was resolved by excluding Alkhouli et al. 2020 (*I* ^2^ = 0%) (Figure S5).

#### 3.5.2 Safety outcomes

**FIGURE 6:**
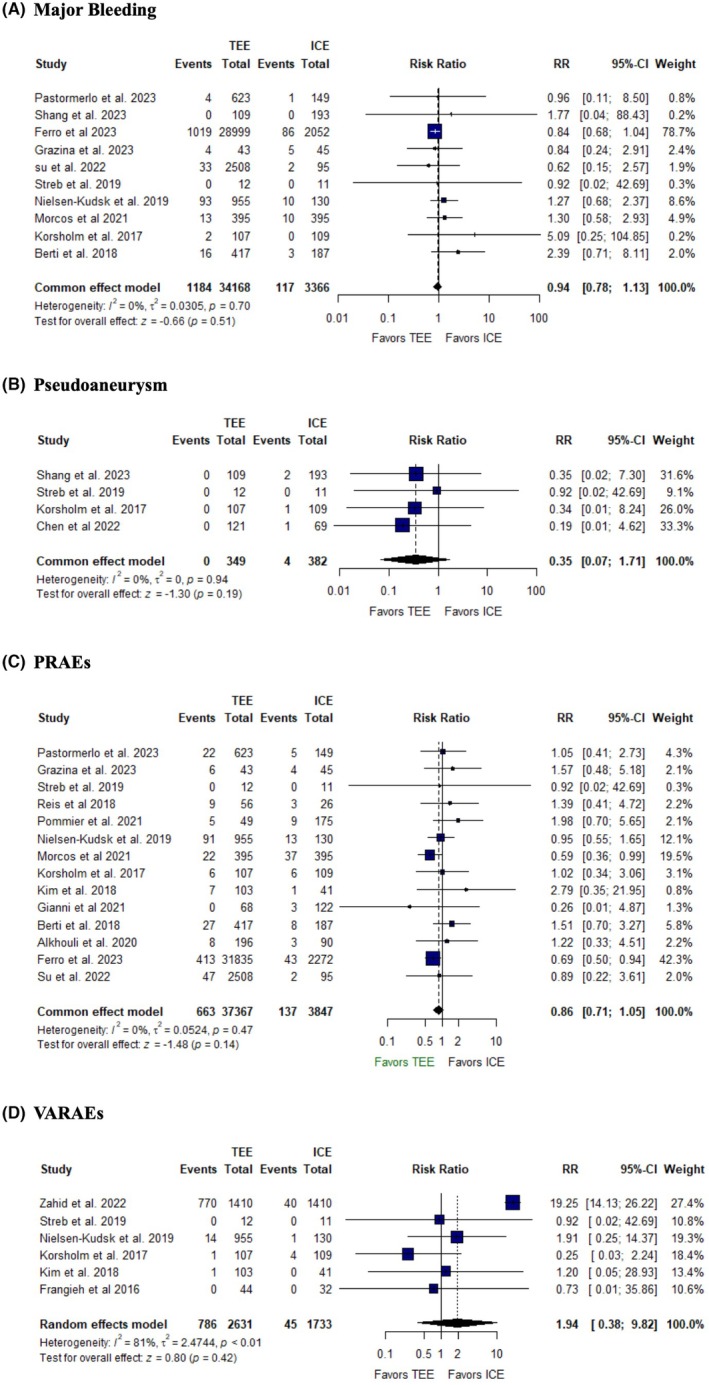
Forest plots of (A) major bleeding, (B) pseudoaneurysm, (C) PRAEs, (D) VARAEs. CI, confidence interval; PRAEs, procedure‐related adverse events; RR, risk ratio; VARAEs, vascular access‐related adverse events.

There was no significant difference between ICE and TEE groups in major bleeding (RR: 0.94, 95% CI: [0.78, 1.13], *p* = 0.51, *I* ^2^ = 0%) (Figure 6A), pseudoaneurysm (RR: 0.35, 95% CI: [0.07, 1.71], *p* = 0.19, *I* ^2^ = 0%) (Figure 6B), procedure related adverse events (PRAEs) (RR: 0.86, 95% CI: [0.71, 1.05], *p* = 0.14, *I* ^2^ = 0%), and vascular access related adverse events (VARAEs) (RR: 1.94, 95% CI: [0.38, 9.82], *p* = 0.42, *I* ^2^ = 81%) (Figure 6C). The studies were homogeneous in all outcomes, except high heterogeneity was observed in VARAEs, which was resolved by removing Zahid et al. 2020 (*I* ^2^ = 0%) (Figure S6).

## 4 DISCUSSION

Our meta‐analysis included 19 observational studies, encompassing 44,706 patients—5,604 in the ICE group and 39,102 in the TEE group, compared across primary, secondary, and safety outcomes. Unlike previous meta‐analyses,^36^, ^37^, ^38^, ^39^, ^40^ our study has incorporated recent clinical studies and expanded the analysis to include additional outcomes, with a particular emphasis on the safety.

ICE demonstrated comparable success rate and all‐cause mortality to TEE. However, ICE was associated with a little high procedure success rate and a greater risk of pericardial effusion. Among secondary outcomes, there were no significant differences in procedure duration, fluoroscopy time, contrast volume, or hospital stay. Notably, patients in the TEE group were more likely to require multiple devices more often, though the average number of devices used did not differ between the two groups. Safety outcomes, including major bleeding, pseudoaneurysm, device embolization, stroke, and transient ischemic attack, showed no significant differences between ICE and TEE. These findings suggest that both ICE and TEE are viable imaging modalities for LAAO, especially in patients not suitable for GA.

AF accounts for approximately 5% of ischemic strokes annually and is linked to high mortality and morbidity.^11^ LAAO serves as an effective stroke prevention strategy for patients ineligible for oral anticoagulants. Intraoperative imaging is crucial, with TEE being the primary modality due to its superior visualization of cardiac anatomy, real‐time complication detection, and ability to delegate imaging tasks to a specialist, allowing the operator to focus on the procedure. However, complications occur in up to 3% of cases, with postprocedural esophageal injury reported in over 80% of patients, particularly those over 65 years old, a demographic commonly undergoing LAAO.^41^, ^42^ Given these risks, ICE is gaining traction as an alternative and is increasingly utilized in structural heart procedures.^43^

In this meta‐analysis, ICE demonstrated a significantly higher procedure success rate than TEE; however, this difference was unlikely to be clinically significant. This finding contrasts with studies by Ferro et al.,^21^ Su et al.,^33^ Nielsen‐Kudsk et al.,^28^ and previous meta‐analyses,^36^, ^37^, ^38^, ^39^, ^40^ all of which found no significant difference between the two modalities. The improved success rate with ICE may be attributed to technological advancements and increased operator experience over time. However, the lack of clinical significance suggests that both ICE and TEE remain viable options for procedural guidance. This is particularly reassuring for operators, as a common concern with ICE‐guided closure is the added responsibility of simultaneously managing imaging and performing the procedure.

However, ICE was significantly associated with a higher risk of pericardial effusion compared to TEE. This finding is consistent with studies by Ferro et al.^21^ and Morcos et al.^27^ but contrasts with those by Pastormerlo et al.,^29^ Zahid et al.,^34^ and Nielsen‐Kudsk et al.,^28^ which reported no significant difference between the two modalities.

The increased risk of pericardial effusion with ICE may stem from complications related to more extensive catheter manipulation.^27^ Unlike atrial septal closure, which involves minimal movement within the right atrium, LAAO requires more complex navigation, including the interatrial septum, left atrial chamber, and occasionally the coronary sinus or left pulmonary artery. This greater degree of manipulation increases the likelihood of pericardial effusion. Additionally, the suboptimal visualization of key structures during ICE‐guided procedures may have contributed to these complications.^27^

This aspect highlights the importance of operator experience, as variations in expertise may influence the outcomes. Ferro et al. specifically linked the increased risk of pericardial effusion to differences in operator experience. In their study, 82% of ICE procedures were performed by operators who had conducted fewer than 10 ICE‐guided implants, which contrasts with outcomes from operators with much more experience using TEE guidance. Interestingly, the rate of pericardial effusion among ICE operators decreased as their experience grew, indicating a learning curve.^21^ Therefore, Physicians adopting ICE for LAAO implantation should be aware of the increased risk of pericardial effusion.

Despite the increased risk of pericardial effusion associated with ICE, our analysis found no significant difference between ICE and TEE in terms of all‐cause mortality and hospital stay. Regarding all‐cause mortality, our findings are consistent with those of Ferro et al.,^21^ Grazina et al.,^23^ Nielsen‐Kudsk et al.,^28^ Morcos et al.,^27^ and Liang et al. meta‐analysis^40^ Who similarly reported no significant differences between the two techniques. This suggests that while ICE may be associated with certain procedural complications, these do not appear to lead to worse overall clinical outcomes.

Regarding hospital stay, our findings are consistent with those of Pastormerlo et al.,^29^ Zahid et al.,^34^ and Korsholm et al.,^26^ all of them reported no significant difference between ICE and TEE. In contrast, Nielsen‐Kudsk et al.,^28^ Alkhouli et al.,^17^ and Frangieh et al.^20^ observed a significant increase in hospital stays with TEE, while Morcos et al.^27^ reported a significant decrease with TEE.

The extended hospital stay in Nielsen‐Kudsk et al., Alkhouli et al., and Frangieh et al. may be attributed to their inclusion of older patients who are more prone to prolonged recovery. Additionally, the higher complication rates reported by Nielsen‐Kudsk et al. and Alkhouli et al. likely contributed to extended hospitalization in these studies. These variations highlight the impact of patient demographics and procedural complications on hospital stay durations, emphasizing the need to consider individual patient characteristics and potential risks when choosing between ICE and TEE for procedural guidance.

Our findings indicate that procedural parameters, including procedure duration, fluoroscopy time, and contrast volume, demonstrated no statistically significant differences between TEE and ICE, consistent with the meta‐analyses of Liang et al., Zhang et al., and Jhand et al.^36^, ^38^, ^40^ However, significant heterogeneity was observed for these outcomes, suggesting that the results should be interpreted cautiously. This heterogeneity likely stems from several factors, including differences in the study definitions, procedural protocols, and operator experience across the included trials. Additionally, the type of occlusion devices employed, varying imaging platforms, and differences in institutional expertise may have further influenced procedural efficiency and fluoroscopy exposure. These factors, combined with patient selection criteria and center‐specific learning curves, could have contributed to the inconsistent findings.

Moreover, our analysis showed that the TEE group required more than one device more often compared to the ICE group; however, there was no significant difference in the mean number of devices used between the two groups. Our findings align with those of Shang et al.^35^ and Gianni et al.,^22^ but contrast with Ferro et al., which reported a significant increase in the number of devices required in the TEE group. The discrepancy between our findings and those of Ferro et al. may result from differences in study design, patient populations, procedural protocols, operator experience, institutional practices, and variations in the criteria for using or replacing multiple devices. Clinically, our findings suggest that ICE improves procedural efficiency by reducing the need for multiple device deployments compared to TEE. The higher device requirement in the TEE group may reflect challenges in achieving optimal positioning due to differences in visualization, maneuverability, or anatomical assessment. Fewer device deployments can shorten procedure time, reduce complexity, lower costs, and minimize complications like endothelial trauma or embolization. These findings highlight ICE's potential to streamline procedures while maintaining safety and efficacy.

Our analysis found no significant difference between TEE and ICE in the risk of device embolization, stroke, major bleeding, pseudoaneurysm, or transient ischemic attack. These results align with prior meta‐analyses by Liang et al.,^40^ Velagapudi et al.,^39^ Diaz et al.,^37^ and Zhang et al.,^38^ emphasizing that both TEE and ICE are comparably safe when performed by skilled operators. This is likely to be due to improvements in procedural techniques, patient management, and device technology, which have contributed to reducing risks associated with both modalities. The consistency of safety outcomes across multiple studies further strengthens the reliability of these findings.

The OPTION trial assessed the safety and efficacy of LAAC following AF ablation in 1600 patients with a CHA_2_DS_2_‐VASc score ≥2 for men and ≥3 for women. Participants were randomized to receive either LAAC using the Watchman device or continued oral anticoagulation therapy. The primary safety endpoint, nonprocedure‐related major or clinically relevant nonmajor bleeding at 36 months, were significantly lower in the LAAC group (8.5%) compared to the anticoagulation group (18.1%). In terms of efficacy, the composite rate of all‐cause mortality, stroke, or systemic embolism was similar between the two groups (5.3% for LAAC vs. 5.8% for anticoagulation), indicating noninferiority. Major bleeding events were also slightly lower in the LAAC group (3.9%) compared to the anticoagulation group (5.0%). These results suggest that LAAC combined with AF ablation reduces bleeding risks without compromising stroke prevention, supporting the potential of ICE‐guided LAAC under conscious sedation in the catheterization lab.^44^

The introduction of three‐dimensional intracardiac echocardiography (3D‐ICE) represents a major advancement in imaging for LAAO. Unlike traditional 2D‐ICE, 3D‐ICE provides real‐time, high‐resolution visualization from multiple cross‐sectional planes, improving accuracy in device sizing, and placement. It shows strong concordance with pre‐procedural TEE measurements and reduces the need for device recapture and repositioning. Notably, 3D‐ICE eliminates the need for esophageal intubation, allowing procedures under conscious sedation, enhancing patient comfort, reducing anesthesia risks, and potentially enabling same‐day discharge.^45^, ^46^

Our meta‐analysis included studies utilizing various ICE devices, including the Watchman, Amplatzer Cardiac Plug (ACP)/Amulet, and LAmbre systems. Differences in design and imaging capabilities among these devices may influence procedural outcomes and safety profiles in LAAO. Some ICE systems provide higher resolution imaging or greater maneuverability, potentially improving device deployment accuracy and reducing complications. However, due to the heterogeneity of ICE devices across the included studies and the absence of subgroup analyses based on specific equipment, we could not evaluate the individual impact of each device. Future research comparing ICE systems in LAAO procedures is needed to clarify how device‐specific features influence procedural success and patient outcomes.

Compared to the recently published meta‐analysis by Serpa et al.,^47^ our study offers several novel contributions that enhance the current understanding of imaging guidance in LAAO procedures. While both studies included a similar number of observational studies and patients, our analysis incorporated a more recent and comprehensive literature search from five databases, yielding a larger pooled cohort of 50,863 patients versus 42,474 in the prior study. We uniquely focused on several clinically relevant secondary outcomes not fully addressed by Serpa et al., such as procedural duration, fluoroscopy time, contrast volume, hospital stay, and the frequency of using more than one device. Importantly, our analysis examined outcomes like device embolization, TIA, and the need for multiple devices in greater detail. Additionally, we performed extensive heterogeneity assessments with leave‐one‐out sensitivity analyses to ensure robustness. By presenting both efficacy and safety outcomes with enhanced granularity and a broader set of procedural metrics, our study provides clinicians with a more nuanced comparison between ICE and TEE guidance in LAAO, which was not captured in the earlier publication.

### 4.1 Strengths and limitations

Our study is the most comprehensive meta‐analysis to date comparing ICE and TEE in LAAO for patients with AF, incorporating 19 observational studies with a total of 50,863 patients. We examined 17 outcomes, with a particular focus on safety, a key aspect that has been overlooked in previous meta‐analyses. Notably, our analysis includes several distinctive endpoints that have not been thoroughly assessed in prior work, such as the need for more than one device, device embolization, and neurological outcomes including stroke and TIA. By addressing these clinically meaningful and underreported complications, our study provides a more granular and robust evaluation of the comparative safety profiles of ICE and TEE, thereby offering added clinical value and filling important gaps in the existing literature.

However, there are several limitations to consider. Firstly, our analysis only included studies published in English. Additionally, some of the studies we reviewed were retrospective, which could introduce a higher risk of bias, especially in patient selection. The variation in resources and the differing levels of expertise among medical practitioners may have significantly influenced treatment decisions, limiting the broader applicability of our findings. There was a significant variation among the inserted devices, and there was not enough data to conduct a subgroup analysis based on the devices used for LAAO. Furthermore, heterogeneity was observed across various outcomes. It is also worth noting that our analysis did not assess outcomes such as quality of life, landing zone, in‐room time, and per‐device leaks, as these were not reported in the studies we included.

### 4.2 Implications of our findings in practice and recommendations for future research

Our findings suggest that both ICE and TEE are viable imaging modalities for LAAO in patients with AF. The comparable success rates and safety profiles of both techniques highlight their clinical utility. However, the higher risk of pericardial effusion associated with ICE ‘warrants consideration’ when selecting the imaging modality. Future research should focus on prospective studies with standardized protocols to explore long‐term outcomes, cost‐effectiveness, and patient‐centered outcomes such as quality of life. Additionally, further investigation into the advantages of each modality for specific patient subgroups would provide valuable insights for clinical decision‐making.

## 5 CONCLUSION

Our meta‐analysis concluded that ICE can be a viable alternative to TEE for guiding LAAO, particularly in patients unsuitable for GA. It can also reduce the need for GA and hence adverse effects and resources associated with it, requires fewer devices, and demonstrates comparable safety and efficacy outcomes, though it may increase the risk of pericardial effusion. Future research should focus on prospective studies that examine long‐term outcomes, cost‐effectiveness, and patient‐centered factors to further guide the selection of imaging modality for LAAO in clinical practice.

## AUTHOR CONTRIBUTIONS

U.K. and A.N. conceived the idea. A.M.A. and J.A. designed the research workflow. M.K.H and M.I. searched the databases. S.R., A.M.A., M.I., and S.H.Z.B. screened the retrieved records. Six reviewers (M.M., M.S., S.R., A.T.A., J.A., M.N.R., and M.H.K) extracted relevant data, while six reviewers A.N., U.K., Z.M., F.H.G., and A.T.A assessed the quality of evidence, and U.K. resolved the conflicts. A.M.A. performed the analysis. A.N. and M.S. wrote the final manuscript. U.K. supervised the project. All authors have read and agreed to the final version of the manuscript.

## FUNDING INFORMATION

We received no funding for this study.

## CONFLICT OF INTEREST STATEMENT

Authors declare no conflict of interests for this article.

## Supporting information


Data S1.
