# Supplementary material for: Intracardiac echocardiography versus transesophageal echocardiography guidance on left atrial appendage occlusion in patients with atrial fibrillation: A systematic review and meta‐analysis
Source: J Arrhythm. 2025 Jul 1;41(4):e70115. doi: 10.1002/joa3.70115 (PMC12209866; doi:10.1002/joa3.70115)
Supplement: Supplementary file 1 — Data S1. [file JOA3-41-e70115-s001.docx]

**Supplementary Materials.**

**Table legend.**

Table S1: Search strategy.

Figures Legends.

Figure S1: Sensitivity analysis in procedure duration.

Figure S2: Sensitivity analysis in fluoroscopy time.

Figure S3: Sensitivity analysis in contrast volume.

Figure S4: Sensitivity analysis in length of hospital stay.

Figure S5: Sensitivity analysis in the mean number of devices.

Figure S6: Sensitivity analysis in VARAEs.

***Search strategy:***

| Database | SS | Search field | Search Results |
| --- | --- | --- | --- |
| PubMed | (“Intracardiac” OR “Transesophageal” OR “Transthoracic”) AND (“Echocardiography” OR “echo” OR “doppler Echo” OR “doppler Echocardiography”) AND (“Left Atrial Appendage” OR “Left Atrial Appendage obstruction” OR “Left Atrial Appendage Occlusion” OR “Left Atrial Appendage Intervention” OR “LAA Closure”) | All fields | 2464 |
| Central | (“Intracardiac” OR “Transesophageal” OR “Transthoracic”) AND (“Echocardiography” OR “echo” OR “doppler Echo” OR “doppler Echocardiography”) AND (“Left Atrial Appendage” OR “Left Atrial Appendage obstruction” OR “Left Atrial Appendage Occlusion” OR “Left Atrial Appendage Intervention” OR “LAA Closure”) |  | 130 |
| Web of Science | (“Intracardiac” OR “Transesophageal” OR “Transthoracic”) AND (“Echocardiography” OR “echo” OR “doppler Echo” OR “doppler Echocardiography”) AND (“Left Atrial Appendage” OR “Left Atrial Appendage obstruction” OR “Left Atrial Appendage Occlusion” OR “Left Atrial Appendage Intervention” OR “LAA Closure”) |  | 2315 |
| Scopus | (“Intracardiac” OR “Transesophageal” OR “Transthoracic”) AND (“Echocardiography” OR “echo” OR “doppler Echo” OR “doppler Echocardiography”) AND (“Left Atrial Appendage” OR “Left Atrial Appendage obstruction” OR “Left Atrial Appendage Occlusion” OR “Left Atrial Appendage Intervention” OR “LAA Closure”) |  | 3944 |
| Embase | (“Intracardiac” OR “Transesophageal” OR “Transthoracic”) AND (“Echocardiography” OR “echo” OR “doppler Echo” OR “doppler Echocardiography”) AND (“Left Atrial Appendage” OR “Left Atrial Appendage obstruction” OR “Left Atrial Appendage Occlusion” OR “Left Atrial Appendage Intervention” OR “LAA Closure”) |  | 6897 |


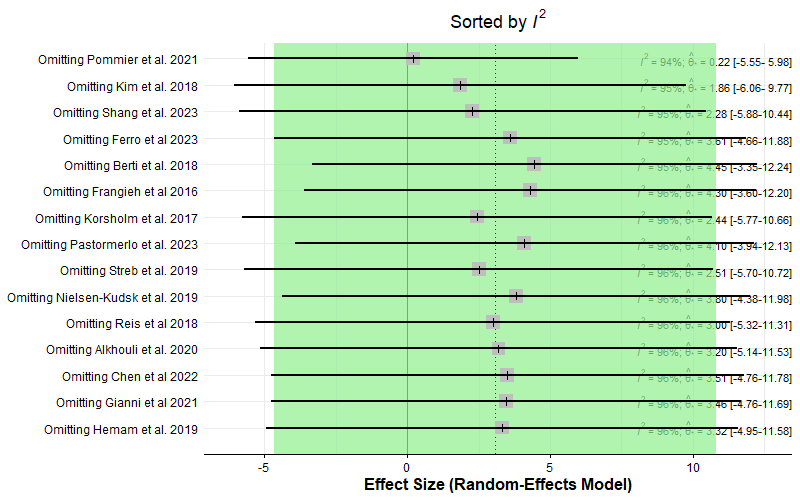


Figure S1 shows sensitivity analysis in procedure duration


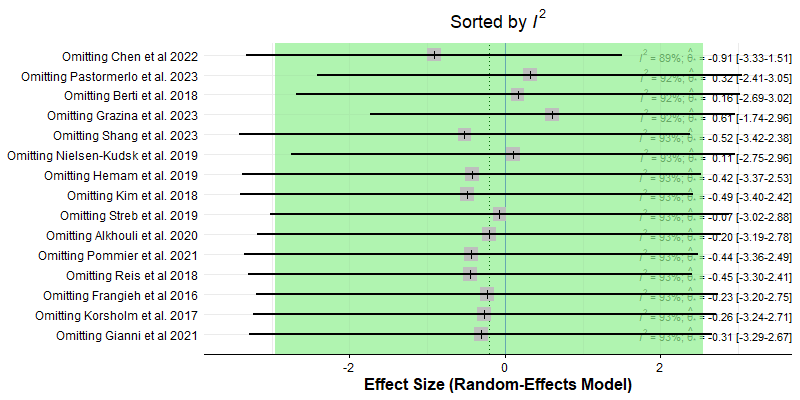


Figure S2 shows sensitivity analysis in fluoroscopy time


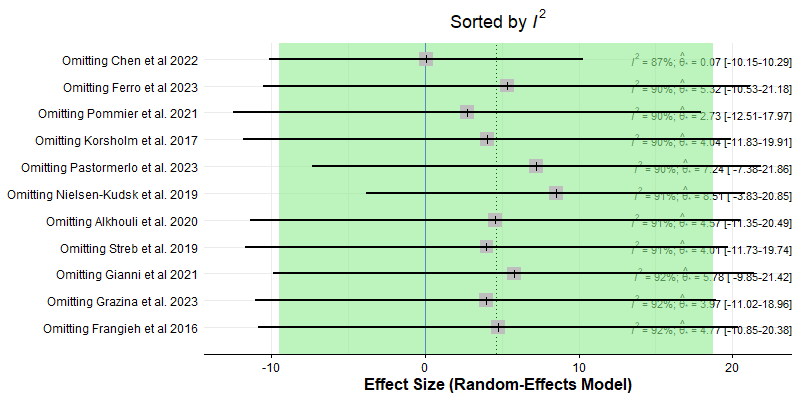


Figure S3 shows sensitivity analysis in contrast volume


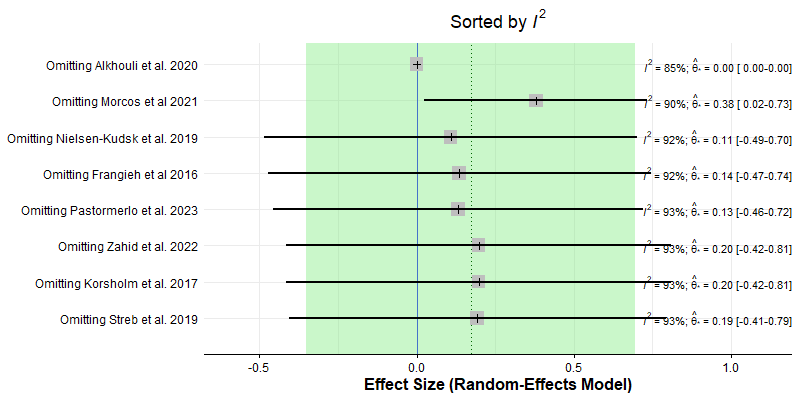


Figure S4 shows sensitivity analysis in length of hospital stay


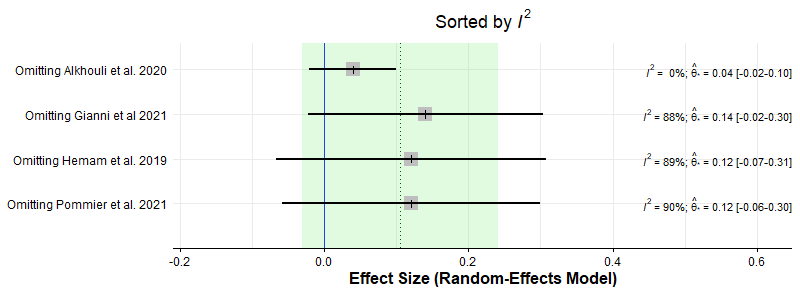


Figure S5 shows sensitivity analysis in the mean number of devices


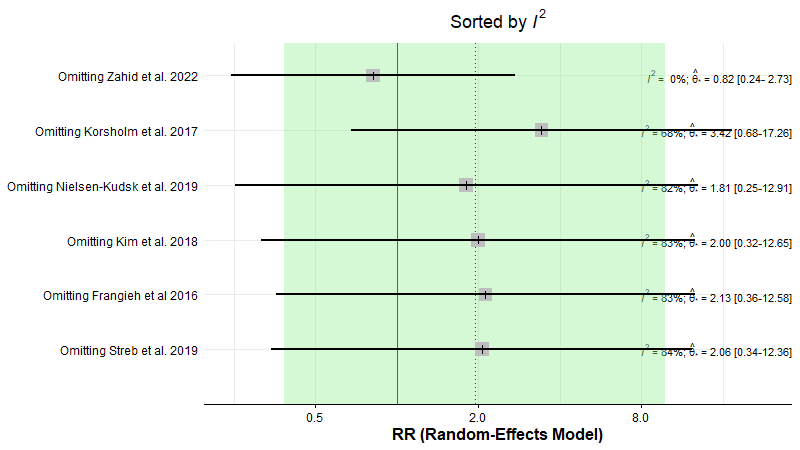


Figure S6 shows sensitivity analysis in VARAEs
